# A Balloon-assisted Colonoscope does not Increase the Adenoma Miss Rate: Outcomes of a Single-center Tandem Randomized Clinical Trial

**DOI:** 10.1055/a-2901-5859

**Published:** 2026-07-24

**Authors:** Jean-Philippe Ratone, Yanis Dahel, Cristophe Zemmour, Solène Hoibian, Christian Pesenti, Florat Poizat, Marc Giovannini, Fabrice Caillol

**Affiliations:** 1 Endoscopy Unit 56181 Paoli-Calmettes InstituteMarseilleProvence-Alpes-Côte d’AzurFrance; 2 Clinical & Innovation Research Direction, Biostastistics & Methodology Unit 56181 Paoli-Calmettes InstituteMarseilleProvence-Alpes-Côte d’AzurFrance; 3 Pathology Unit 56181 Paoli-Calmettes InstituteMarseilleProvence-Alpes-Côte d’AzurFrance

**Keywords:** screening colonoscopy, adenoma miss rate, adenoma detection rate

## Abstract

**Background** Colorectal cancer is the third leading cause of cancer death worldwide. Colonoscopy can prevent this complication but is imperfect because of missed adenoma. A device involving a variable-rigidity balloon has been developed: G-EYE colonoscopy (GEYEC). Only a few prospective studies on GEYEC are available. We compared GEYEC to standard colonoscopy (SC) during screening colonoscopy.

**Methods** We performed a single-center back-to-back colonoscopy tandem randomized clinical trial. Patients older than 50 years were referred for screening colonoscopy, among whom one-third with a positive fecal immunochemical test were included. In Arm A, patients underwent SC followed by GEYEC; in Arm B, the opposite occurred. The primary objective was to assess and compare adenoma miss rate (AMR) between arms. The secondary objectives were to compare advanced AMRs (aAMRs), adverse events for each colonoscope and withdrawal stability rates for GEYEC.

**Results** One hundred ninety-five patients were included between January 2020 and June 2023. A total of 164 patients (83 women, 51%) completed the two procedures, performed by six experienced operators. The mean AMRs were 0.438 in Arm A and 0.393 in Arm B ( *p* = 0.713). The mean aAMRs were 0.176 in Arm A and 0.036 in Arm B ( *p* = 0.76). The rate of significant adverse events (≥AGREE II) was 1.8%, and none were attributed to the GEYEC balloon. In 79% of cases, operators felt that GEYEC provided more stability during withdrawal.

**Conclusion** Although it is reliable and safe, compared with SC, GEYEC does not reduce AMR or aAMR. However, it increases the comfort of scope withdrawal during screening.

## 1 Introduction

Despite improvements in prevention and treatment, colorectal cancer remains a major public health problem worldwide (third leading cause of cancer and second leading cause of cancer-related death worldwide). 1 Colonoscopy has long been recognized as an excellent means of prevention and significantly reduces the rate of cancer in monitored populations. 2 3 However, some patients develop colorectal cancer between two colonoscopies, indicating that prevention can still be improved. 4 Quality indicators have been developed to increase the adenoma detection rate (ADR), which is associated with less interval colorectal cancer (ICC). 5 The adenoma miss rate (AMR) and ADR are indicators that are statistically correlated with ICC. Several devices or techniques have been developed to improve this detection rate, with encouraging results. 6 In clinical practice, the techniques used are often those that do not change the colonoscopy technique. Indeed, despite their efficacy, promising techniques such as Endocuff have seen their development hampered by their financial cost, their sometimes delicate use or even their ecological cost. 7 Other interesting techniques, such as FUSE, have disappeared due to buyouts between companies. 8 9 Over the past 5 years, artificial intelligence (AI) has proven to be effective among experts and nonexperts and is easily usable. 10 11 However, its effectiveness concerns mainly small adenomas, and it does not a priori allow us to see blind areas, for example, those behind folds. Although this approach will become a general standard very soon, the gastroenterologist will always focus on improving the blind zones suspected to be responsible for ICC, particularly in the right colon. G-EYE colonoscopy (GEYEC), developed by SMART MEDICAL, is one of the techniques whose aim is to increase the visualization of blind areas without changing the colonoscopy technique. This balloon-assisted colonoscopy consists of performing classical colonoscopy and withdrawing it with the balloon inflated. The balloon is integrated with a classical colonoscope and does not change the insertion time. In addition, GEYEC can be combined with AI. A randomized clinical trial revealed an improvement in ADR by GEYE and a greater detection of advanced adenoma. More serrated lesions and flat adenomas were detected by GEYEC in the right colon. 12 Preliminary studies also supported the use of GEYEC. 13 14 15 To our knowledge, only one tandem randomized clinical trial has compared GEYEC and standard colonoscopy. However, this was a pilot study with a relatively limited number of patients and the AMR with the standard colonoscope was particularly low (7.5%) in a slightly different population (≥40 years). Therefore, these encouraging results require confirmation. A tandem back-to-back colonoscopy is an excellent design to avoid subjectivity. We wanted to perform a tandem randomized study to answer the following question: is the rate of missed adenoma significantly different between procedures using GEYEC and standard forward-viewing colonoscopy?

## 2 Materials and Methods

### 2.1 Study Design and Patients

We performed a monocentric tandem randomized colonoscopy trial in Marseille, France, at Paoli-Calmettes Institute between January 2020 and May 2023. We enrolled patients aged ≥50 years referred for diagnostic colonoscopy.

We excluded patients with genetic diseases that promote colorectal adenomas (such as polyposis, Lynch, etc.), those who underwent colonic surgery, those with inflammatory bowel disease, those with coagulation disorders preventing a colonoscopy from being performed, and patients referred to our center for colorectal endoscopic resection. We also excluded second-time patients with a bowel preparation score <7 and/or with one segment <2.

### 2.2 Randomization

Patients were randomized (1:1) following a permutation random block size procedure stratified on the basis of fecal immunochemical test (FIT)-positive status between the initial consultation in which the procedure was explained and the procedure itself. In Arm A, standard colonoscopy was performed first, and GEYEC was performed first in Arm B.

### 2.3 Procedures

All procedures were performed under sedation with propofol without orotracheal intubation except if the anesthesiologist found some criteria for orotracheal intubation. We performed a same-day back-to-back colonoscopy to assess AMRs. In Arm A, the patient’s referring operator performed a standard colonoscopy with a Pentax colonoscope, and then a second operator from the team performed a second colonoscopy at the same time with a Pentax colonoscope equipped with the G-EYE system (Smart Medical, Israel). In Arm B, the referring operator performed a colonoscopy using the G-EYE colonoscope, and then a second operator performed a second colonoscopy using a standard PENTAX colonoscope (EC38i10F2). A preliminary clinical study and an internal service analysis confirmed that the operators’ ADRs were comparable. 9 All polyps were removed at each colonoscopy except for small typical hyperplastic polyps in the rectosigmoid location (left in place at the operator’s discretion). All polyps were analyzed by the local pathology unit and categorized according to segment and procedure into adenoma-serrated, hyperplastic, or others. In the case of adenomas, those with high-grade dysplasia (HGD), intramucosal adenocarcinoma or up to 10 mm in size were considered advanced adenomas. CO _2_ insufflation was used for all procedures. At the end of the procedure, the operator was required to answer a binary question (yes/no) to determine whether the G-EYE had provided better stability and comfort during colonoscopy withdrawal.

All the colonoscopies were performed by six endoscopists experienced in diagnosis (at least 4 years) and in therapeutic endoscopy (at least 2 years in therapeutic endoscopy piecemeal EMR, ERCP, EUS, and therapeutic-EUS). Only three of the six endoscopists had experience in endoscopic submucosal resection. GEYEC had been used by all the team for 1 year. The quality criteria for screening colonoscopy included a 6-minute withdrawal time and bowel preparation. 16 Patients with a bowel preparation scale score of less than 7 or with a segment of less than or equal to 2 were excluded. 17

Polyps were removed according to the ESGE guidelines of that time with a cold snare for sizes up to 9 mm and by endoscopic mucosal resection for sizes ≥10 mm. Diminutive polyps (<3 mm) could be removed using cold forceps at the discretion of the operator. 18

Patients taking antiplatelet and anticoagulation medications were not excluded. We managed the patients according to the ESGE guidelines. 19

### 2.4 Definition

The AMR of the standard colonoscope corresponds to the rate of adenomas detected during the second procedure in Arm A. Conversely, the AMR of GEYEC corresponds to the rate of adenomas detected during the second procedure in arm B.

Advanced AMR (aAMR) revealed intramucosal adenocarcinoma, HGD adenoma, adenoma up to 1 cm in size or serrated up to 1 cm.

The ADR corresponds to the proportion of patients over 50 years old in whom at least one adenoma is identified, relative to the total number of colonoscopies performed. Thus, the ADR for the standard colonoscope corresponds to the ADR of the first procedures in Arm A, and the ADR for the GEYEC corresponds to the ADR of the first procedures in arm B.

### 2.5 Endpoints

The primary endpoints were the mean AMR of each colonoscope (standard and GEYEC) and the percentage of patients with at least one missed adenoma in each arm. The secondary endpoints were as follows:

Mean aAMR for each colonoscope, ADR for standard colonoscopy and GEYEC, ADR for men and women, mean AMR and ADR for FIT+ patients, and stability with the GEYEC during the withdrawal and adverse events by type of colonoscope.

### 2.6 Statistical Analysis

All the statistical analyses were performed at a significance level of α=0.05 (unless otherwise specified) using SAS 9.4 software. Patient and treatment characteristics were described using counts (percentages) for qualitative endpoints and medians [ranges] for quantitative endpoints. Analyses were performed following the intent-to-treat principle, that is, by considering the patients in the arm allocated by the randomization.

The sample size was determined at 80% power by a classic unilateral Z test for a 15% difference between the arm percentages of patients with at least one missed adenoma, more precisely by assuming respective expected rates of 20% in Arm A and 5% in Arm B in accordance with the rates reported in the literature. 20 21 Anticipating 20% nonevaluable patients, a total of 196 patients were planned to be selected. Following the recommendations of Song et al., this sample size permitted us to sequentially test the null hypothesis of equal detection rates in the overall population and then in the FIT+ subpopulation using a two-step procedure while controlling the global significance level α=0.05. 22 The specified significance levels for each planned test and global methodology are detailed in Supplementary File 1. Bilateral 90%-level Wald confidence intervals (CIs) of these rates were computed (to determine the lowest boundary rate that would be detected with a 5% right-sided procedure).

The mean AMR and aAMR were estimated using a generalized linear model with a logarithmic link function, negative binomial distribution and randomization arm as independent covariates. Asymptotic bilateral CIs for these rates and their differences were computed.

Exact Fisher’s tests were used to compare the ADR and assistance for stability provided by the GEYEC rates of the two arms.

### 2.7 Legal and Ethical Considerations

All patients were informed at the initial consultation and gave their oral and written consent. The national medical research safety authorities and ethics committees have validated the protocol. The Paoli-Calmettes Institute was the sponsor. This clinical trial is registered at ClinicalTrials.gov (number NCT03961893).

## 3 Results

### 3.1 Patient Characteristics

**Fig. 1:**
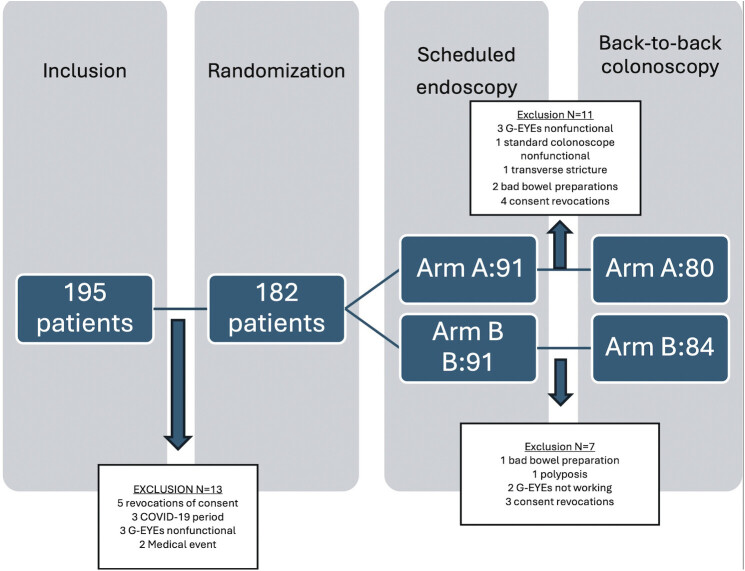
Flowchart. *COVID-19: Coronavirus disease 2019. G-EYE: colonoscope equipped with a G-EYE device. ARM A = standard colonoscopy following by G-EYE colonoscopy. ARM B = G-EYE colonoscopy following by standard colonoscopy.*

One hundred ninety-five patients were included between January 2020 and June 2023. Thirty-one patients were excluded from the analysis (nonoptimal preparation, material not available, patient wishes, and trial suspension during the COVID-19 period). The study flowchart is shown in Fig. 1 . The median patient age was 64 years (range [50–82]). A total of 164 (83 women, 51%) underwent the two procedures. Fifty-two patients had a positive FIT, and 112 underwent a colonoscopy for other indications; the patient characteristics and main colonoscopy indications are shown in Table 1 .

**Table 1:** Patient characteristics.

Characteristic	All ( *N* = 164)	Arm A ( *N* = 84)	Arm B ( *N* = 80)
Age (years), median [range]	64 [50–82]	63 [50–82]	66 [50–79]
Sex			
*Female*	83 (50.6%)	38 (45.2%)	45 (56.3%)
*Male*	81 (49.4%)	46 (54.8%)	35 (43.7%)
Main colonoscopy indication			
*Symptoms*	64 (39%)	32 (38.1%)	32 (40%)
*Personal and familial history of CCR and adenomas*	46 (28.1%)	25 (29.8%)	21 (26.2%)
*PET–CT fixation*	2 (1.2%)	0 (0%)	2 (2.5%)
*FIT +*	52 (31.7%)	27 (32.1%)	25 (31.3%)

*PET–CT: Positron emission tomography–computed tomography (PET–CT)* .

### 3.2 Primary Endpoints: AMR by Standard Colonoscopy and GEYEC and Percentage of Patients with at Least One Missed Adenoma

The estimated mean AMRs were 0.438 (95% CI [0.261, 0.614]) for standard colonoscopy (Arm A) and 0.393 [0.232, 0.553] for the GEYEC (Arm B). There was no cancer among the missed adenomas. No significant difference between the two groups was found (estimated difference = −0.045 [−0.283, 0.194], *p* = 0.713). The results regarding the primary endpoint are summarized in Table 2 .

**Table 2:** Primary endpoint results.

Contrast	Estimate mean AMR	Confidence interval	*p*
Arm A	0.438	[0.261, 0.614]	
Arm B	0.393	[0.232, 0.553]	
Delta B–A	−0.045	[−0.283, 0.194]	0.713

*AMR: Adenoma miss rate* .

In Arm A (standard colonoscopy first), 24 patients (30%) had at least one adenoma miss that was secondarily identified via GEYEC. In Arm B, 24 patients (28.6%) had at least one adenoma miss that was secondarily identified by the standard colonoscope. The difference between those two rates was not significant following the procedure detailed in Supplementary File 1 (weighted *Z* test statistic *Z*_1_ = 0.183, *p* = 0.427). Characteristics of missed adenomas are shown in Table 5 .

### 3.3 Secondary Endpoints

#### 3.3.1 Advanced Miss Rate Adenoma (aAMR) by Standard Colonoscopy and GEYEC

The estimated mean aAMRs were 0.1 (95% CI [0.015, 0.185]) for standard colonoscopy (Arm A) and 0.036 [0, 0.08] for GEYEC (Arm B). There was no significant difference between the two arms (estimated difference = −0.064 [−0.16, 0.031], *p* = 0.176). These advanced adenomas corresponded exclusively to 11 adenomas or serrated lesions up to 1 cm in nine patients. No intramucosal adenocarcinoma or HGD was identified. The aAMR results are listed in Table 3 .

**Table 3:** Advanced adenoma miss rate.

Contrast	Estimate mean aAMR	Confidence interval	*p*
Arm A	0.1	[0.015, 0.185]	
Arm B	0.036	[0, 0.08]	
Delta B–A	−0.064	[−0.16, 0.031]	0.176

*aAMR: Advanced adenoma miss rate* .

#### 3.3.2 ADR for Each Colonoscope (Standard Colonoscopy and GEYEC) According to Sex

The ADRs for standard colonoscopy (Arm A, procedure 1) were 51% (90% CI, [42%–60%]) for standard colonoscopy and 54% (45–63%) for GEYEC (Arm B, procedure 1). There was no significant difference between these two rates ( *p* = 0.876).

For men and women, the ADRs were, respectively, 60% (46–74%) and 44% (32%–57%) in Arm A and 59% (47–71%) and 47% (34%–61%) in Arm B, respectively. The results are shown in Table 4 .

**Table 4:** Outcomes regarding adenoma detection rate (ADR).

Adenoma detection rate (ADR)	Male ( *N* = 81)		Female ( *N* = 83)		All ( *N* = 164)	
	Sta. Col. ( *N* = 35)	GEYEC ( *N* = 46)	Sta. Col. ( *N* = 45)	GEYEC ( *N* = 38)	Sta. Col. ( *N* = 80)	GEYEC ( *N* = 84)
No.	21	27	20	18	41	45
Rate [90%-level Wald bilateral CI]	60% [46%–74%]	59% [47%–71%]	44% [32%–57%]	47% [34%–61%]	51% [42%–60%]	54% [45%–63%]

CI: Confidence Interval.

*Sta. Col.: standard colonoscopy; GEYEC: G-EYE colonoscopy* .

**Table 5:** Characteristics of missed adenomas.

	Patients with ≥ 1 MA ( *n* / *N* )	Total MA ( *n* )	Right colon MA ( *n* )	Transverse colon MA ( *n* )	Left colon MA ( *n* )	Rectum MA ( *n* )	Missed serrated adenoma ( *n* )	Missed low-grade dysplasia adenoma ( *n* )	Missed high-grade dysplasia ( *n* )	Missed low-grade adenoma >1 cm ( *n* )
Arms A	24/80	32	15	10	6	1	9	15	0	8
Arm B	24/84	33	14	11	7	1	5	25	0	3

*MA: Missed Adenomas* .

#### 3.3.3 Results for the FIT-positive Subgroup

In this subgroup, the AMRs were 6/25 (24%, 90% CI [10–38%]) for standard colonoscopy (Arm A) and 7/29 (26% [12–40%]) for GEYEC (Arm B). The ADR rates for FIT+ patients were 52% (13/25) for standard colonoscopy (Arm A) and 67% (18/27) for GEYEC (Arm B).

#### 3.3.4 Adverse Events According to the AGREE Classification and Perforation Rate by Each Colonoscope

AGREE classification was used to describe adverse events, and grade ≥ II was considered to indicate significant adverse events. 23

No diagnosis perforation occurred during standard colonoscopy or GEYEC (0%).

One perforation (0.6%, 1/164) after endoscopic mucosal resection was constated by procedure 2 but occurred during procedure 1 in Arm A and was managed by endoscopy (clips) in procedure 2.

One case of significant delayed bleeding occurred (0.6%, 1/164) and required rehospitalization and a hemostatic endoscopic procedure. Resection was performed with a standard colonoscopy in Arm A.

These two adverse events were classified as grade IIIA.

One patient (0.6%, 1/164) in Arm B with a single lung had severe respiratory decompensation and was hospitalized in the intensive care unit for 5 days (grade IVa).

The rate of significant adverse events (≥AGREE II) was 1.8% (3/164 patients), with two grade IIIA and one grade IV events. None of the complications were considered to be specifically due to the use of the GEYEC or its distal balloon system.

#### 3.3.5 GEYEC Stability and Comfort during the Withdrawal

The operators reported that the G-EYE improved stability and comfort during withdrawal in 79% of cases.

## 4 Discussion

Our tandem randomized clinical trial did not confirm the outcomes of the previous studies using the GEYEC. The mean AMR was similar despite the study design being powered to detect a 15% difference. Unfortunately, the results were also the same for the subgroup with advanced adenomas.

This trial has several strengths. First, the population was representative of a classical screening population with patients older than 50 years, including one-third FIT-positive patients. Second, the ADR was high, and the percentage of patients with at least one adenoma missed was approximately 30%. Although the number of patients was inferior to that in the study of Shiffrin et al., the back-to-back design removed any subjectivity from the results. 12 Moreover, the operator switch between the two colonoscopies removed slightly more subjectivity because it caused a limited competition effect.

Another tandem back-to-back study was published by Halpern et al. in 2025. 13 In contrast to our findings, its findings were in favor of GEYEC with only 4.7% missed adenomas compared with 44.7% missed adenomas with a standard colonoscope. In our opinion, the AMR in this study seems to be higher than expected. To explain this discrepancy, one could mention the fact that keeping the same operator between procedures could have unconsciously influenced the result if the belief was strong in the device. The nonblinded nature of most back-to-back colonoscopy studies may also explain the difference in missed adenomas. The habit of using GEYEC cannot be retained, as the study could have had it 2 years before the start of the trial. Our study, like others, revealed that despite the efforts of physicians and industry, there is still significant room for improvement in reducing AMR and the interval cancer rate.

Our study confirms certain points regarding GEYEC. There was no additional morbidity linked to the use of the distal balloon, as no complications specifically linked to GEYEC were noted. The rate of significant complications (AGREE III and above) is not negligible (1.8%), which is due to our decision to not exclude patients on anticoagulants, unlike other studies on GEYEC. 12 This further underscores the safety of the device. Although this is a subjective criterion, operators reported that GEYEC exhibited increased stability and comfort of colonoscope withdrawal (in 79% of cases). Our results validate the use of the GEYEC for screening. Indeed, the ADRs obtained by the GEYEC method in the standard population (54%) and in FIT-positive patients (67%) were superior to the guidelines (≥35% and ≥50%, respectively). 24

However, our trial exhibits several limitations. This was a single-center study. The study design, made to compare AMRs, was too weak to compare ADRs, which is the gold standard. Another potential limitation is the higher-than-expected AMR, which may have affected the study’s statistical power. However, the back-to-back tandem design, with its inherently high detection of missed adenomas, should have been sufficient to reveal any clinically significant differences between the techniques. In addition, given the results and the competitive effect we observed, having two different operators could be considered a limitation by certain experts. Nevertheless, the experience level of the team is homogeneous, with six diagnosis and therapeutic endoscopists, none of whom are novices. The study, whose idea dates back to 2016, was conducted without AI to avoid distorting the results, as AI was not available in our unit at the time, unlike today. In 2025, we might wonder what the results would be like with AI assistance.

In conclusion, the G-EYE colonoscope is safe and efficient and does not lead to specific adverse events. Even if the withdrawal seems to be more stable, GEYEC does not increase the AMR.

## Supplementary Material

Ergänzendes Material Supplementary Material

## References

[1] Sung H Ferlay J Siegel R L Global cancer statistics 2020: GLOBOCAN estimates of incidence and mortality worldwide for 36 cancers in 185 countries CA Cancer J Clin 2021 71 03 209 249 33538338 10.3322/caac.21660

[2] Zauber A G Winawer S J O'Brien M J Colonoscopic polypectomy and long-term prevention of colorectal-cancer deaths N Engl J Med 2012 366 08 687 696 22356322 10.1056/NEJMoa1100370 PMC3322371

[3] Nishihara R Wu K Lochhead P Long-term colorectal-cancer incidence and mortality after lower endoscopy N Engl J Med 2013 369 12 1095 1105 24047059 10.1056/NEJMoa1301969 PMC3840160

[4] Corley D A Jensen C D Marks A R Adenoma detection rate and risk of colorectal cancer and death N Engl J Med 2014 370 14 1298 1306 24693890 10.1056/NEJMoa1309086 PMC4036494

[5] Rex D K Schoenfeld P S Cohen J Quality indicators for colonoscopy Gastrointest Endosc 2015 81 01 31 53 25480100 10.1016/j.gie.2014.07.058

[6] Spadaccini M Schiliro A Sharma P Repici A Hassan C Voza A Adenoma detection rate in colonoscopy: how can it be improved? Expert Rev Gastroenterol Hepatol 2023 17 11 1089 1099 37869781 10.1080/17474124.2023.2273990

[7] Li A Yang J Z Yang X X Endocuff-assisted colonoscopy versus cap-assisted colonoscopy for adenoma detection rate: a meta-analysis of randomized controlled trials J Gastroenterol Hepatol 2020 35 12 2066 2073 32562282 10.1111/jgh.15155

[8] Gralnek I M Siersema P D Halpern Z Standard forward-viewing colonoscopy versus full-spectrum endoscopy: an international, multicentre, randomised, tandem colonoscopy trial Lancet Oncol 2014 15 03 353 360 24560453 10.1016/S1470-2045(14)70020-8 PMC4062184

[9] Ratone J P Bories E Caillol F Impact of full Spectrum endoscopy(R) (Fuse(R), EndoChoice(R)) on adenoma detection: a prospective French pilot study Ann Gastroenterol 2017 30 05 512 517 28845106 10.20524/aog.2017.0176 PMC5566771

[10] Wei M T Shankar U Parvin R Evaluation of computer-aided detection during colonoscopy in the community (AI-SEE): a Multicenter randomized clinical trial Am J Gastroenterol 2023 118 10 1841 1847 36892545 10.14309/ajg.0000000000002239

[11] Karsenti D Tharsis G Perrot B Effect of real-time computer-aided detection of colorectal adenoma in routine colonoscopy (COLO-GENIUS): a single-Centre randomised controlled trial Lancet Gastroenterol Hepatol 2023 8 08 726 734 37269872 10.1016/S2468-1253(23)00104-8

[12] Shirin H Shpak B Epshtein J G-EYE colonoscopy is superior to standard colonoscopy for increasing adenoma detection rate: an international randomized controlled trial (with videos) Gastrointest Endosc 2019 89 03 545 553 30273591 10.1016/j.gie.2018.09.028

[13] Halpern Z Gross S A Gralnek I M Comparison of adenoma detection and miss rates between a novel balloon colonoscope and standard colonoscopy: a randomized tandem study Endoscopy 2015 47 03 238 244 25704662 10.1055/s-0034-1391437

[14] Kiesslich R Teubner D Hoffman A Rey J W Retrospective comparison of G-EYE balloon-colonoscopy with standard colonoscopy for increased adenoma detection rate and reduced polyp removal time Endosc Int Open 2023 11 09 E920 E927 37810901 10.1055/a-2005-6934 PMC10558257

[15] Rey J W Dumcke S Haschemi JG-EYE advanced colonoscopy for improved polyp detection rates – a randomized tandem pilot study with different endoscopists. Z Gastroenterol2018 10.1055/s-0043-124089 29426056

[16] Shaukat A Kahi C J Burke C A Rabeneck L Sauer B G Rex D K ACG clinical guidelines: colorectal cancer screening 2021 Am J Gastroenterol 2021 116 03 458 479 33657038 10.14309/ajg.0000000000001122

[17] Lai E J Calderwood A H Doros G Fix O K Jacobson B C The Boston bowel preparation scale: a valid and reliable instrument for colonoscopy-oriented research Gastrointest Endosc 2009 69(3 Pt 2)620 625 19136102 10.1016/j.gie.2008.05.057 PMC2763922

[18] Ferlitsch M Moss A Hassan C Colorectal polypectomy and endoscopic mucosal resection (EMR): European Society of Gastrointestinal Endoscopy (ESGE) clinical guideline Endoscopy 2017 49 03 270 297 28212588 10.1055/s-0043-102569

[19] Veitch A M Radaelli F Alikhan R Endoscopy in patients on antiplatelet or anticoagulant therapy: British Society of Gastroenterology (BSG) and European Society of Gastrointestinal Endoscopy (ESGE) guideline update Endoscopy 2021 53 09 947 969 34359080 10.1055/a-1547-2282 PMC8390296

[20] van Rijn J C Reitsma J B Stoker J Bossuyt P M van Deventer S J Dekker E Polyp miss rate determined by tandem colonoscopy: a systematic review Am J Gastroenterol 2006 101 02 343 350 16454841 10.1111/j.1572-0241.2006.00390.x

[21] Heresbach D Barrioz T Lapalus M G Miss rate for colorectal neoplastic polyps: a prospective multicenter study of back-to-back video colonoscopies Endoscopy 2008 40 04 284 290 18389446 10.1055/s-2007-995618

[22] Song Y Chi G Y A method for testing a prespecified subgroup in clinical trials Stat Med 2007 26 19 3535 3549 17266164 10.1002/sim.2825

[23] Nass K J Zwager L W van der Vlugt M Novel classification for adverse events in GI endoscopy: the AGREE classification Gastrointest Endosc 2022 95 06 1078 85 e8 34890695 10.1016/j.gie.2021.11.038

[24] Rex D K Anderson J C Butterly L F Quality indicators for colonoscopy Gastrointest Endosc 2024 100 03 352 381 39177519 10.1016/j.gie.2024.04.2905 PMC13192241

