# Supplementary material for: A Balloon-assisted Colonoscope does not Increase the Adenoma Miss Rate: Outcomes of a Single-center Tandem Randomized Clinical Trial
Source: Endosc Int Open. 2026 Jul 24;14:a29015859. doi: 10.1055/a-2901-5859 (PMC13460302; doi:10.1055/a-2901-5859)
Supplement: Supplementary file 1 [file 10-1055-a-2901-5859_29123301.pdf]

Supplementary file: A balloon-assisted colonoscope does not increase the adenoma miss rate: Outcomes of a single-center tandem randomized clinical trial.

Ratone JP, Dahel Y, Zemmour C, Hoibian S, Pesenti C, Poizat F, Giovannini M, Caillol F

Null hypothesis of no difference between the arm rates of patients with at least one missed adenoma was planned to be sequentially tested both in the overall population of randomized patients (H_{01}) and in the FIT+ subpopulation (H_{02}), with a 2-step multiple comparison procedure (Song and Chi 2007, doi: 10.1002/sim.2825).

Global significance level was set to $\alpha = 0.05$ and all following tests were right-sided with alternative hypotheses like: $\pi_A - \pi_B > 0$, where π_A and π_B are respectively the arm A and B missed adenoma rates.

Step 1: Test for joint hypothesis $H_{012} = H_{01} \cap H_{02}$

Let :

- k be the proportion of FIT+ patients
- Z_2 be the Z-test statistic for the null hypothesis H_{02} of no difference between the missed adenoma rates in the FIT+ subpopulation
- $Z_2^{'}$ be the Z-test statistic for the null hypothesis $H_{02}^{'}$ of no difference between the missed adenoma rates in the no-FIT+ subpopulation

We define the weighted Z-test statistic $Z_1 := \sqrt{k}\, Z_2 + \sqrt{1 - k}\, Z_2^{'}$.

Under H_{012}, Z_2, $Z_2^{'}$ and Z_1 statistics follow a standard normal distribution. Let p_1 be the p-value associated with Z_1, p_2 be the p-value associated with Z_2, and assuming the following significance levels as defined in the protocol $\alpha_1 = 0.04$, $\alpha_1^* = 0.20$ and $\alpha_2 = 0.052$, then the test procedure for the hypothesis H_{012} is performed as following:

- If $p_1 \leq \alpha_1$ then H_{012} is rejected
- If $p_1 > \alpha_1^*$ then H_{012} is not rejected
- If $\alpha_1 < p_1 \leq \alpha_1^*$ then consider the analysis with Z_2 and p_2:
    - If $p_2 \leq \alpha_2$ then H_{012} is rejected
    - If $p_2 > \alpha_2$ then H_{012} is not rejected

If the null hypothesis H_{012} is rejected, then proceed to the step 2.

Step 2: Test for single hypotheses H_{01} *and* H_{02}

Each of the hypotheses is tested at the significance level $\alpha = 0.05$. Let $\widetilde{Z_1}$ be the Z-test statistic for H_{01} and $\widetilde{p_1}$ the associated p-value (following a standard normal distribution), then the procedure for testing H_{01} and H_{02} is performed as following:

- If $\widetilde{p_1} \leq \alpha$ then H_{01} is rejected
- If $p_2 \leq \alpha$ then H_{02} is rejected

Supplementary Figure 1 displays the testing procedure process. Red lines highlight the final decision taken based on the results obtained: the joint hypothesis was not rejected at the step 1 ($Z_1 = 0.183$, $p = 0.427$).

Supplementary Figure 1: Testing procedure process. Red lines highlight the final decision taken based on the results obtained: the joint hypothesis was not rejected at the step 1 (Z_1=0.183, p=0.427).

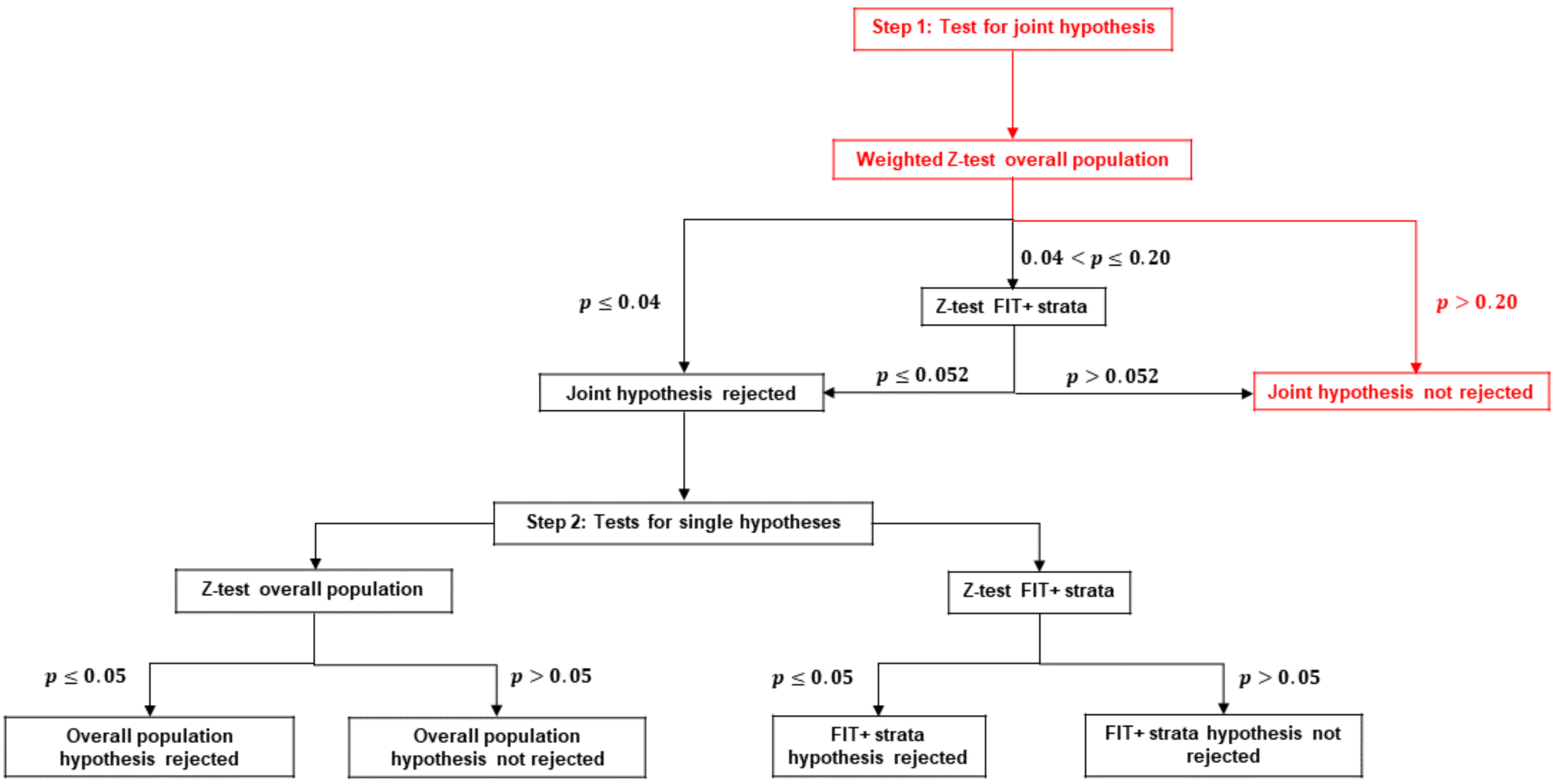

Endosc Int Open | DOI 10.1055/a-2901-5859 | © The Author(s).
